# Things We Do for No Reason—Polymerase Chain Reaction for Lyme Disease Diagnostic Testing

**DOI:** 10.1093/ofid/ofag700

**Published:** 2026-10-30

**Authors:** Wesley D Kufel, Alicia A Zimmer, Rahul Mahapatra, Kristopher M Paolino

**Affiliations:** Department of Pharmacy Practice, State University of New York at Binghamton School of Pharmacy and Pharmaceutical Sciences, Binghamton, New York, USA; Department of Medicine, Division of Infectious Diseases, State University of New York Upstate Medical University, Syracuse, New York, USA; Department of Medicine, Division of Infectious Diseases, State University of New York Upstate Medical University, Syracuse, New York, USA; Department of Medicine, Division of Infectious Diseases, State University of New York Upstate Medical University, Syracuse, New York, USA; Department of Medicine, Division of Infectious Diseases, State University of New York Upstate Medical University, Syracuse, New York, USA

**Keywords:** *Borrelia burgdorferi*, diagnostic stewardship, Lyme disease, polymerase chain reaction

## Abstract

*Borrelia burgdorferi* polymerase chain reaction (PCR) testing demonstrated a low positivity rate of 1.9% (17/885), minimal impact on clinical decision-making, and considerable healthcare cost expenditures in a Lyme disease endemic area. These findings reinforce adherence to guideline-recommended serologic testing and support diagnostic stewardship initiatives to limit unnecessary PCR utilization.

Lyme disease, caused by the spirochete bacterium *Borrelia burgdorferi*, is currently the most common vector-borne disease with nearly half a million new cases reported each year in the United States [1–4]. More specifically in New York State, the reported incidence of Lyme disease increased from 37.2 to 164.8 cases per 100,000 residents between 2000 and 2024 [5]. Thus, early recognition of the clinical manifestations and accurate detection of *B. burgdorferi* are important to help guide clinical decision-making [1]. Furthermore, initiation of prompt treatment is needed to help prevent potential progression to disseminated disease (e.g., Lyme neuroborreliosis, Lyme carditis, Lyme arthritis) and/or symptom sequelae following treatment of Lyme disease [1]. While early localized Lyme disease is diagnosed clinically, laboratory testing is generally needed for the diagnosis of disseminated disease [1]. Commercially available diagnostic tests include indirect detection methods to measure the host's response to infection (i.e., serologic testing) or direct detection methods for the presence of *B. burgdorferi* bacteria (e.g., polymerase chain reaction (PCR)) [6, 7].

Currently, modified two-tiered serologic testing (mTTT) or standard two-tiered serologic testing is the guideline-recommended diagnostic in combination with clinical features, patient history, and risk factors rather than direct detection methods such as PCR [1]. This is because two-tiered serologic testing has high sensitivity (70-100%) in patients with common extracutaneous manifestations that develop weeks to months after initial infection and high specificity (>95%) when performed and interpreted appropriately according to current guidelines [1, 8–10]. However, two-tiered serologic testing still has important limitations. For seropositive patients, it can be difficult to determine whether antibody reactivity is due to past infection versus currently active infection. For seronegative patients, there is a chance of false negativity in the first days to weeks following initial exposure as a detectable antibody response can take weeks to months to develop [1, 11].

Given some of these practical challenges and limitations of serologic testing, clinicians may opt to perform alternative laboratory diagnostic methods such as direct detection via PCR in addition to or in place of serologic testing to help assess the potential diagnosis of Lyme disease. However, *B. burgdorferi* PCR testing has significant limitations that clinicians should be aware of. First, the sensitivity of *B. burgdorferi* PCR testing varies by specimen source [5]. Synovial fluid has the highest sensitivity (50-100%) as compared to PCRs of the blood and cerebrospinal fluid (CSF), which have much lower sensitivity (∼5-17%) [1, 6, 8, 12–16]. Specificity from each of these specimens ranges from ∼95-100%. The low sensitivity reflects the sparseness of spirochete bacteria components in these clinical specimens, and therefore, a negative PCR from these specimen sources has limited diagnostic utility [17]. Second, PCR testing detects genetic material of an organism and is unable to discern between viable and unviable material. Therefore, a positive PCR result alone does not confirm an active infection since a PCR can remain positive for weeks to months following appropriate treatment and may lead to subsequent unnecessary treatment courses [1]. Third, there are no currently FDA-cleared molecular assays for *B. burgdorferi* PCR testing, and therefore, testing is typically available only at large reference laboratories, which significantly delays turnaround time [1].

Clinicians in both endemic and non-endemic areas including those without infectious diseases (ID) training and experience may not be aware of the significant limitations of *B. burgdorferi* PCR testing. This could potentially lead to untoward consequences for clinical decision-making including unnecessary *B. burgdorferi* PCR testing, delayed or missed alternative diagnoses, and a considerable financial burden to both the healthcare system and patients [18, 19].

At our institution, anecdotal reports of many non-ID clinicians performing PCR testing were common for diagnostic evaluation of Lyme disease. We contend that *B. burgdorferi* PCR testing should not be included in Lyme disease diagnostic workups for the aforementioned reasons and may represent a potential target for diagnostic stewardship intervention as more data are needed in this space. Therefore, we sought to evaluate the clinical utility of *B. burgdorferi* PCR testing and assess the clinical and financial impact on patient care management in a Lyme disease endemic area of Upstate New York.

## METHODS

This was a single-center, retrospective cohort study of all *B. burgdorferi* PCR results from January 1, 2024-June 30, 2026. The study was performed at the State University of New York Upstate University Hospital, a 752-bed, tertiary care, academic medical center located in Syracuse, New York, which serves 17 counties in Upstate New York. The PCR results during this study period were extracted from the electronic medical record via the laboratory report function in Epic and included collection date, specimen type, and qualitative result. All completed *B. burgdorferi* PCR tests were included for evaluation. Tests that were ordered but were cancelled or not performed prior to processing/sending out were excluded. The *B. burgdorferi* PCR, available for providers to order as ‘Lyme PCR fluid (send out)’ in the electronic medical record, is a send out laboratory test to LabCorp (Burlington, NC, USA) and the cost during the study period was $454 including send out laboratory fees. Of note, our institution performs mTTT in house.

The primary outcome was the positivity rate of the *B. burgdorferi* PCR. Secondary outcomes included the number of tests performed, positive tests impacting management, and cost. This study was deemed exempt by the Institutional Review Board at SUNY Upstate Medical University. Descriptive statistics were used to characterize results.

## RESULTS

Between January 1, 2024-June 30, 2026, there were 885 *B. burgdorferi* PCR tests performed. Primary and secondary outcome data are displayed in Table 1. Most specimens were from CSF (50.1%, 443/885) followed by synovial fluid (35.6%, 315/885) and blood (14.4%, 127/885). For the primary outcome, the positivity rate from any specimen type was 1.9% (17/885). Furthermore, the positivity rate was 5.4% (17/315) for synovial fluid whereas no PCR tests were positive on either the CSF or blood specimens. The total cost of the *B. burgdorferi* PCR tests performed was $401,790. More specifically, the cost was $201,122, $143,010, and $57,658 for CSF, synovial fluid, and blood specimens, respectively. Additionally, the number of PCRs performed, number of positive PCRs, positivity rate, and cost by specimen source for each year included in the study period can be found in the Supplementary Appendix (Tables S1-S3).

**Table 1.** Number of PCRs Performed, Number of Positive PCRs, Positivity Rate, and Cost by Specimen Source

Specimen Source	Number of PCRs Performed, N (%)	Number of Positive PCRs	Positivity Rate (%)	Cost^a^ ($)
**Cerebrospinal fluid**	443 (50.1)	0	0	201,122
**Synovial fluid**	315 (35.6)	17	5.4	143,010
**Blood**	127 (14.4)	0	0	57,658
**Total**	**885** (**100)**	**17**	**1.9**	**401,790**

^a^Based on a cost of $454 per PCR.

Of the 17 positive *B. burgdorferi* PCRs, 11 (64.7%) did not impact management since treatment was already initiated based on mITT positive results and clinical presentation before PCR testing resulted (Table 2). The median time from collection to resulted value was 7.2 (IQR 1.9) days given the send out turnaround time. Therefore, six positive PCRs (36.3%) assisted with clinical decision-making for Lyme disease management; however, five did not have mTTT performed at the time of PCR testing. Of the 17 positive PCRs, ID was consulted in three cases.

**Table 2.** Impact of Positive *Borrelia burgdorferi* PCR testing on Diagnosis and/or Treatment

Patient	Prior PCR	MTTT Result	Lyme Diagnosis	Impact of PCR On Diagnosis And/or Treatment Comments
1	No	Yes IgM – IgG +	Lyme arthritis of L knee	No mTTT resulted 13 days before PCR resulted (ordered after mTTT). Prescribed doxycycline based on mTTT
2	No	Yes IgM – IgG +	Lyme arthritis of R knee	No mTTT resulted 8 days before PCR resulted. Prescribed doxycycline based on mTTT
3	No	Yes IgM – IgG +	Lyme arthritis of L knee	No mTTT resulted 7 days before PCR resulted. Prescribed doxycycline based on mTTT
4	No	Yes IgM – IgG +	Lyme arthritis of L knee	No mTTT resulted 9 days before PCR resulted. Prescribed doxycycline based on mTTT
5	Yes (Negative ∼1 and 3 years prior)	Yes IgM – IgG +	Lyme arthritis of L knee	No mTTT resulted 8 days before PCR resulted. Prescribed doxycycline based on mTTT
6	No	Yes IgM – IgG +	Lyme arthritis of L knee	No mTTT resulted 4 days before PCR resulted. Prescribed doxycycline based on mTTT
7	No	Yes IgM – IgG +	Lyme arthritis of L knee	No mTTT resulted 5 days before PCR resulted. Prescribed doxycycline based on mTTT Outpatient ID consult
8	No	Yes IgM + IgG+	Lyme arthritis of L knee	No mTTT resulted 8 days before PCR resulted. Prescribed doxycycline based on mTTT
9	No	Yes IgM + IgG +	Lyme arthritis of L knee	No mTTT resulted 9 days before PCR resulted. Prescribed doxycycline based on mTTT
10	No	Yes IgM + IgG +	Lyme arthritis of L knee	No mTTT resulted 5 days before PCR resulted. Prescribed doxycycline based on mTTT
11	No	Yes IgM + IgG +	Lyme arthritis of L knee	No mTTT resulted 4 days before PCR resulted. Prescribed doxycycline based on mTTT
12	No	Not performed	Lyme arthritis of L knee	Yes Doxycycline prescribed based on PCR result No mTTT performed Outpatient ID consult
13	No	Not performed	Lyme arthritis of R knee	Yes Doxycycline prescribed based on PCR result No mTTT performed Outpatient ID consult
14	No	Not performed	Lyme arthritis of R knee	Yes Doxycycline prescribed based on PCR result No mTTT performed
15	No	Not performed	Lyme arthritis of R knee	Yes Doxycycline prescribed based on PCR result No mTTT performed
16	Yes (negative ∼ 3 years prior)	Yes IgM – IgG +	Lyme arthritis of L knee	Yes Initial presentation was not thought to be consistent with Lyme disease and doxycycline was not prescribed until PCR resulted
17	No	Not performed	Lyme arthritis of L knee	Yes Doxycycline prescribed based on PCR result No mTTT performed

Abbreviations: ID, infectious diseases; L, left; mTTT, modified two-tiered testing; PCR, polymerase chain reaction; R, right.

## DISCUSSION

Among this cohort of patients over a 2.5-year period in a Lyme endemic area, *B. burgdorferi* PCR testing had a considerably low positivity rate of 1.9% (17/885) despite substantial utilization. Furthermore, positive PCRs only resulted from synovial fluid specimens and the positivity rate for this specimen in this cohort was also low at 5.4% (17/315). Additionally, 11 of the 17 positive PCRs did not influence management because treatment had been initiated based on the already available mTTT results. Among patients with a positive PCR and known mTTT results, all PCR-positive patients were also seropositive, which further questions the diagnostic utility of the PCR. In five out of six positive PCRs, mTTT was not performed, and it is unclear how useful the PCR would have been if the mTTT was performed in these cases. While the sensitivity of the *B. burgdorferi* PCR is higher on synovial fluid, there is considerable variability in the sensitivity range (50-100%) based on currently available data [1, 6, 8, 12–16].

Another important finding was that no CSF or blood specimens yielded a positive result despite these specimen types accounting for nearly two-thirds of all tests performed and the low sensitivity (∼5-17%) on these specimens [1, 6, 8, 13–15]. Aside from the limited clinical utility, the total cost for *B. burgdorferi* PCR testing was $401,790 over the 2.5-year period representing a substantial cost burden to both the health system and patients overall [19]. Because the *B. burgdorferi* PCR is a send out test that takes multiple days for results to return (median 7.2 days) with considerable cost expenditures and negligible influence on diagnosis and treatment decision-making, *B. burgdorferi* PCR is not helpful in most clinical situations representing low diagnostic utility [6]. Tests with limited clinical utility should not be used if the result will not impact clinical management [20, 21]. These findings highlight an important diagnostic stewardship opportunity to reduce unnecessary PCR utilization while providing clinician education on promoting adherence to evidence-based diagnostic recommendations for Lyme disease.

Based on these findings, our health system prioritized diagnostic stewardship interventions to remove *B. burgdorferi* PCR tests from the orderable database and only allow ordering through a “miscellaneous laboratory test” approach to mitigate unnecessary use. Furthermore, ID clinicians will provide targeted education for clinicians in emergency medicine, neurology, orthopedics, cardiology, rheumatology, and primary care on the limitations and low diagnostic utility of the *B. burgdorferi* PCR.

Strengths of this study include large-scale, real-world patient data from a Lyme disease endemic area over a 2.5-year period. Nonetheless, this study is not without limitations. First, our findings may not be generalizable to other health systems because this was a single-center study. Second, clinical decision-making was assessed through documentation in the electronic medical record, and we were unable to determine clinicians’ reasoning for performing PCR testing. Third, we did not evaluate the potential downstream and untoward consequences of negative PCR testing, including delays in alternative diagnoses or additional healthcare utilization. Lastly, the study design did not allow for a true calculation of diagnostic sensitivity for the *B. burgdorferi* PCR because the mTTT results for the PCR negative cohort were not collected and not every patient in the PCR positive cohort had mTTT performed. That said, coupling seropositivity with a positive PCR still does not conclusively establish that a patient has active Lyme disease as this still requires clinical signs and symptoms consistent with Lyme disease. Furthermore, previous studies have already evaluated the diagnostic sensitivity of the *B. burgdorferi* PCR on blood, CSF, and synovial fluid [1, 6, 8, 12–16].

Our data strongly dissuade use of the *B. burgdorferi* PCR for blood and CSF, as none of these samples yielded positive results. Likewise, most patients with positive synovial fluid PCRs already had diagnostic serologic evidence of Lyme disease before PCR results became available, demonstrating little incremental diagnostic benefit beyond guideline-recommended two-tiered serologic testing. The prolonged turnaround time identified in our study further diminished its clinical value because treatment decisions had already been made in most patients before results became available. In the few patients that the PCR altered management, most had not undergone mTTT before PCR ordering, suggesting that deviations from established and guideline-recommended diagnostic algorithms accounted for the perceived utility of PCR. Given the additional healthcare costs of this diagnostic test of low utility, clinicians should avoid *B. burgdorferi* PCR testing. We hope our reported data increases awareness for diagnostic stewardship interventions to mitigate *B. burgdorferi* PCR testing and associated healthcare expenditures, especially as Lyme disease incidence increases.

## Supplementary Material

ofag700_Supplementary_Data

## Supplementary Data

Supplementary materials are available at *Open Forum Infectious Diseases* online. Consisting of data provided by the authors to benefit the reader, the posted materials are not copyedited and are the sole responsibility of the authors, so questions or comments should be addressed to the corresponding author.

## References

[1] Lantos PM, Rumbaugh J, Bockenstedt LK, et al Clinical practice guidelines by the Infectious Diseases Society of America (IDSA), American academy of neurology (AAN), and American college of rheumatology (ACR): 2020 guidelines for the prevention, diagnosis and treatment of Lyme disease. Clin Infect Dis 2021; 72:e1–e48. 10.1093/cid/ciaa1215 33417672

[2] Kugeler KJ, Schwartz AM, Delorey MJ, Mead PS, Hinckley AF. Estimating the frequency of Lyme disease diagnoses, United States, 2010-2018. Emerg Infect Dis 2021; 27:616–9. 10.3201/eid2702.202731 PMC7853543 33496229

[3] Hinckley AF, Connally NP, Meek JI, et al Lyme disease testing by large commercial laboratories in the United States. Clin Infect Dis 2014; 59:676–81. 10.1093/cid/ciu397 PMC4646413 24879782

[4] Kugeler KJ, Earley A, Mead PS, Hinckley AF. Surveillance for Lyme disease after implementation of a revised case definition—united States, 2022. MMWR Morb Mortal Wkly Rep 2024; 73:118–23. 10.15585/mmwr.mm7306a1 PMC10899080 38358952

[5] New York State Department of Health Bureau of Communicable Disease Control . *Tick-Borne Disease Surveillance Report, 2025. Available at:* https://www.health.ny.gov/diseases/communicable/lyme/docs/tbd_report_2025.pdf. Accessed September 16, 2026.

[6] Nigrovic LE, Lewander DP, Balamuth F, et al The Lyme disease polymerase chain reaction test has low sensitivity. Vector Borne Zoonotic Dis 2020; 20:310–3. 10.1089/vbz.2019.2547 31821110

[7] Schutzer SE, Body BA, Boyle J, et al Direct diagnostic tests for Lyme disease. Clin Infect Dis 2019; 68:1052–7. 10.1093/cid/ciy614 PMC6399434 30307486

[8] Moore A, Nelson C, Molins C, Mead P, Schriefer M. Current guidelines, common clinical pitfalls, and future directions for laboratory diagnosis of Lyme disease, United States. Emerg Infect Dis 2016; 22:1169–77. 10.3201/eid2207.151694 PMC4918152 27314832

[9] Molins CR, Delorey MJ, Sexton C, Schriefer ME. Lyme borreliosis serology: performance of several commonly used laboratory diagnostic tests and a large resource panel of well-characterized patient samples. J Clin Microbiol 2016; 54:2726–34. 10.1128/JCM.00874-16 PMC5078550 27558183

[10] Steere AC, McHugh G, Damle N, Sikand VK. Prospective study of serologic tests for Lyme disease. Clin Infect Dis 2008; 47:188–95. 10.1086/589242 PMC5538270 18532885

[11] Aguero-Rosenfeld ME, Nowakowski J, Bittker S, Cooper D, Nadelman RB, Wormser GP. Evolution of the serologic response to Borrelia burgdorferi in treated patients with culture-confirmed erythema migrans. J Clin Microbiol 1996; 34:1–9. 10.1128/jcm.34.1.1-9.1996 PMC228718 8748261

[12] Persing DH, Rutledge BJ, Rys PN, et al Target imbalance: disparity of Borrelia burgdorferi genetic material in synovial fluid from Lyme arthritis patients. J Infect Dis 1994; 169:668–72. 10.1093/infdis/169.3.668 8158048

[13] Ružić-Sabljić E, Cerar T. Progress in the molecular diagnosis of Lyme disease. Expert Rev Mol Diagn 2017; 17:19–30. 10.1080/14737159.2016.1246959 27892737

[14] Dumler JS . Molecular diagnosis of Lyme disease: review and meta-analysis. Mol Diagn 2001; 6:1–11. 10.1054/modi.2001.21898 11257206

[15] Avery RA, Frank G, Eppes SC. Diagnostic utility of Borrelia burgdorferi cerebrospinal fluid polymerase chain reaction in children with Lyme meningitis. Pediatr Infect Dis J 2005; 24:705–8. 10.1097/01.inf.0000172903.14077.4c 16094225

[16] Nocton JJ, Dressler F, Rutledge BJ, Rys PN, Persing DH, Steere AC. Detection of Borrelia burgdorferi DNA by polymerase chain reaction in synovial fluid from patients with Lyme arthritis. N Engl J Med 1994; 330:229–34. 10.1056/NEJM199401273300401 8272083

[17] Aguero-Rosenfeld ME, Wang G, Schwartz I, Wormser GP. Diagnosis of Lyme borreliosis. Clin Microbiol Rev 2005; 18:484–509. 10.1128/CMR.18.3.484-509.2005 PMC1195970 16020686

[18] Kobayashi T, Higgins Y, Melia MT, Auwaerter PG. Mistaken identity: many diagnoses are frequently misattributed to Lyme disease. Am J Med 2022; 135:503–11.e5. 10.1016/j.amjmed.2021.10.040 34861197

[19] Yu H, Fee RM, Campfield BT, et al Lyme disease and health care costs. JAMA Netw Open 2026; 9:e2552518. 10.1001/jamanetworkopen.2025.52518 PMC12805444 41533379

[20] Davis MR, McCreary EK, Trzebucki AM. Things we do for no reason—ordering streptococcus pneumoniae urinary antigen in patients with community-acquired pneumonia. Open Forum Infect Dis 2024; 11:ofae089. 10.1093/ofid/ofae089 PMC10959547 38524231

[21] Claeys KC, Coffey KC, Morgan DJ. What is diagnostic stewardship? J Appl Lab Med 2025; 10:130–9. 10.1093/jalm/jfae130 39749441

