# Supplementary material for: Things We Do for No Reason—Polymerase Chain Reaction for Lyme Disease Diagnostic Testing
Source: Open Forum Infect Dis. 2026 Oct 30;13(11):ofag700. doi: 10.1093/ofid/ofag700 (PMC13750177; doi:10.1093/ofid/ofag700)
Supplement: Supplementary file 1 [file ofag700_supplementary_data.docx]

**Supplementary Appendix**

**Table S1.** Number of PCRs performed, number of positive PCRs, positivity rate, and cost by specimen source (2024).

| **Specimen source** | **Number of PCRs obtained, n (%)** | **Number of positive PCRs** | **Positivity rate (%)** | **Cost^a^ ($)** |
| --- | --- | --- | --- | --- |
| **Cerebrospinal fluid** | 194 (45.6) | 0 | 0 | 88,076 |
| **Synovial fluid** | 157 (36.9) | 11 | 7.0 | 71,278 |
| **Blood** | 74 (17.4) | 0 | 0 | 33,596 |
| **Total** | **425 (100)** | **11** | **2.6** | **192,950** |

^a^Based on a cost of $454 per PCR.

**Table S2.** Number of PCRs performed, number of positive PCRs, positivity rate, and cost by specimen source (2025).

| **Specimen source** | **Number of PCRs obtained, n (%)** | **Number of positive PCRs** | **Positivity rate (%)** | **Cost^a^ ($)** |
| --- | --- | --- | --- | --- |
| **Cerebrospinal fluid** | 171 (53.9) | 0 | 0 | 77,634 |
| **Synovial fluid** | 112 (35.3) | 5 | 4.5 | 50,848 |
| **Blood** | 34 (10.7) | 0 | 0 | 15,436 |
| **Total** | **317 (100)** | **5** | **1.6** | **143,918** |

^a^Based on a cost of $454 per PCR.

**Table S3.** Number of PCRs performed, number of positive PCRs, positivity rate, and cost by specimen source (2026).

| **Specimen source** | **Number of PCRs obtained, n (%)** | **Number of positive PCRs** | **Positivity rate (%)** | **Cost^a^ ($)** |
| --- | --- | --- | --- | --- |
| **Cerebrospinal fluid** | 78 (54.5) | 0 | 0 | 35,412 |
| **Synovial fluid** | 46 (32.2) | 1 | 2.2 | 20,884 |
| **Blood** | 19 (13.3) | 0 | 0 | 8,626 |
| **Total** | **143 (100)** | **1** | **0.7** | **64,922** |

^a^Based on a cost of $454 per PCR.
